# The interplay between malaria vectors and human activity accounts for high residual malaria transmission in a Burkina Faso village with universal ITN coverage

**DOI:** 10.1186/s13071-023-05710-7

**Published:** 2023-03-15

**Authors:** Eleonora Perugini, Wamdaogo M. Guelbeogo, Federica Guglielmo, Cristiana Poggi, Eugenio Gabrieli, Hilary Ranson, Alessandra della Torre, Marco Pombi

**Affiliations:** 1 Department of Public Health and Infectious Diseases, Sapienza University, Rome, Italy; 2 Centre National de Recherche et Formation sur le Paludisme, Ouagadougou, Burkina Faso; 3 Department of Vector Biology, Liverpool School of Tropical Medicine, Liverpool, UK

**Keywords:** *Anopheles coluzzii*, *Anopheles arabiensis*, Biting rhythms, Human exposure, Residual malaria transmission, Entomological inoculation rate

## Abstract

**Background:**

Mosquito and human behaviour interaction is a key determinant of the maximum level of protection against malaria that can be provided by insecticide-treated nets (ITNs). Nevertheless, scant literature focuses on this interaction, overlooking a fundamental factor for efficient malaria control. This study aims to estimate malaria transmission risk in a Burkina Faso village by integrating vector biting rhythms with some key information about human habits.

**Methods:**

Indoor/outdoor human landing catches were conducted for 16 h (16:00–08:00) during 8 nights (September 2020) in Goden village. A survey about net usage and sleeping patterns was submitted to half the households (October–December 2020). A subsample of collected specimens of *Anopheles gambiae* sensu lato was molecularly processed for species identification, *Plasmodium* detection from heads-thoraxes and L1014F pyrethroid-resistance allele genotyping. Hourly mosquito abundance was statistically assessed by GLM/GAM, and the entomological inoculation rate (EIR) was corrected for the actual ITN usage retrieved from the questionnaire.

**Results:**

Malaria transmission was mainly driven by *Anopheles coluzzii* (68.7%) followed by *A. arabiensis* (26.2%). The overall sporozoite rate was 2% with L1014F estimated frequency of 0.68 (*N* = 1070 out of 15,201 *A. gambiae* s.l. collected). No major shift in mosquito biting rhythms in response to ITN or differences between indoor and outdoor catches were detected. Impressive high biting pressure (mean 30.3 mosquitoes/person/hour) was exerted from 20:00 to 06:00 with a peak at 4:00. Human survey revealed that nearly all inhabitants were awake before 20:00 and after 7:00 and at least 8.7% had no access to bednets. Adjusting for anthropological data, the EIR dropped from 6.7 to 1.2 infective bites/person/16 h. In a scenario of full net coverage and accounting only for the human sleeping patterns, the daily malaria transmission risk not targetable by ITNs was 0.69 infective bites.

**Conclusions:**

The high mosquito densities and interplay between human/vector activities means that an estimated 10% of residual malaria transmission cannot be prevented by ITNs in the village. Locally tailored studies, like the current one, are essential to explore the heterogeneity of human exposure to infective bites and, consequently, to instruct the adoption of new vector control tools strengthening individual and community protection.

**Graphical Abstract:**

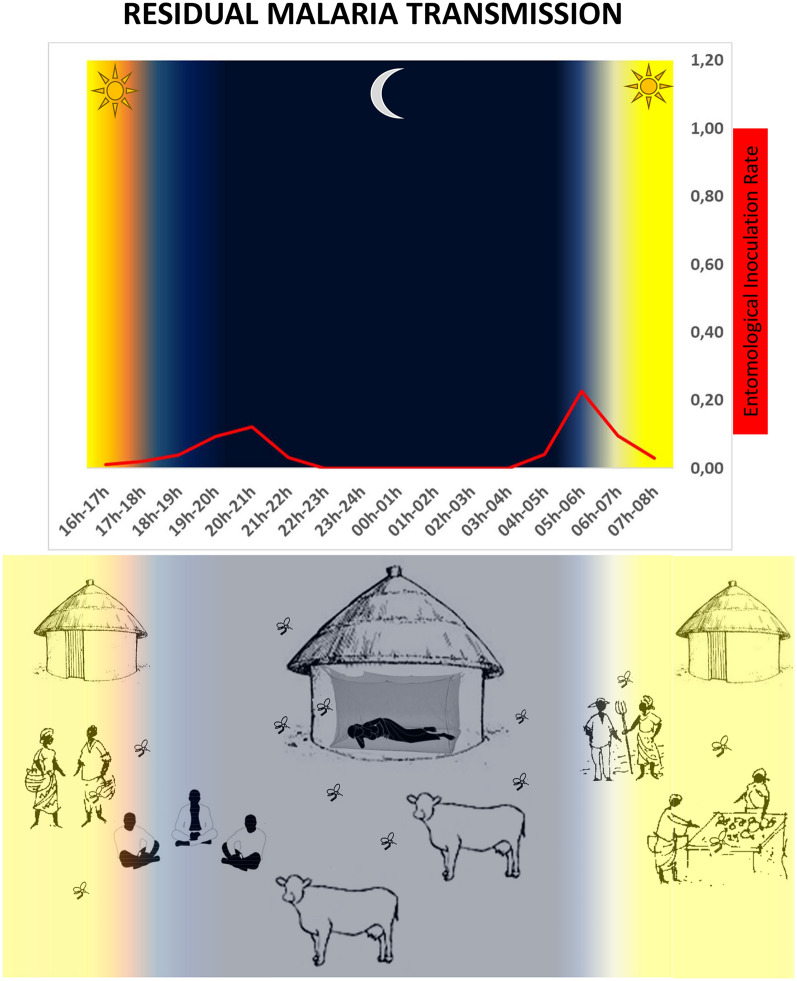

**Supplementary Information:**

The online version contains supplementary material available at 10.1186/s13071-023-05710-7.

## Background

The World Health Organisation (WHO) recommends the use of insecticide-treated nets (ITNs) for malaria vector control. The current WHO policy aims to reach “universal coverage” in endemic countries—a benchmarked 80% net access/usage in each community—through cyclic 3-year mass distribution campaigns [1–3]. It has been estimated that in sub-Saharan countries, which account for 95% of the global malaria burden, ITNs contributed 68% of the 663 million prevented clinical cases from 2000 to 2015 in Africa [4, 5]. This achievement relies on the combination of individual and community protection exerted by the physical and chemical barrier of the nets, which hampers vectors’ human blood-feeding behaviour: major African malaria vectors are anthropophilic species with a biting peak mostly coinciding with human sleeping hours (i.e. central hours of the night) [6–12].

Despite ITNs' incontestable success, their effectiveness in sub-Saharan Africa is heterogeneous. There are 10 countries where a stall in the progress against malaria has been registered since 2015, and disease incidence remains very high despite the large bed net coverage [5, 13–18].

Among many causal factors for this scenario, a crucial role is played by vector insecticide resistance, which undermines ITNs’ community protection by reducing mosquito exposure to lethal doses of the pyrethroids in net fibres. Indeed, many physiological resistance mechanisms have been reported in all major African malaria vectors: target-site mutation (e.g. L1014F mutation), increased metabolic detoxification activity/efficacy (e.g. *P450 monooxygenases*), cuticular thickening or changing in hydrocarbon content and binding/sequestration mechanisms [19–30]. Furthermore, some vector species exhibit different forms of behavioural resistance (or behavioural plasticity) comprising changes in biting behaviour that facilitate the avoidance of the insecticidal barrier, such as flexibility in the spectrum of host choice (opportunistic behaviour) and/or blood feeding at places and times when humans are less likely to be protected by nets (i.e. outdoors and at dusk/dawn) [21, 31–33].

There is a limited understanding of the extent and impact of behavioural resistance among major vector species in sub-Saharan countries. Most reports focus on East Africa and mainly refer to *Anopheles arabiensis* (with very little evidence for *Anopheles gambiae* sensu stricto and *A. funestus*); information about West African vector species is currently incomplete [12, 33–52]. Considering that changes in biting behaviour can increase human-vector contact, an efficient malaria control strategy must consider both entomological and anthropological factors and how they interact. In fact, the interplay between mosquito and human behaviour is considered one of the main causes of residual malaria transmission [53, 54], defined as a persistent parasite transmission despite a fully operational net coverage [54–56]. Recent systematic reviews [12, 57] highlighted sparse published data on human activities during the mosquito biting period with only few field reports pairing mosquitoes and human behaviour. The lack of attention to the degree of overlap between mosquito activity and human exposure to bites may affect the accuracy of ITN efficacy estimates. This interaction deserves further investigations as in different settings it can contribute to undermining the protection of ITNs.

Against this background, our study area—the village of Goden in central Burkina Faso—represents an interesting field setting for two major reasons. First, Goden is a rural area severely affected by malaria, like many areas of Burkina Faso, which is among the countries with the highest malaria burden despite mass ITN implementation. Second, we have data on local vectors’ infectivity, behaviour and insecticide resistance over the 10 years of ITN usage in the village [39, 58, 59]. In the context of the present study, we aimed to investigate possible factors sustaining malaria transmission risk in the village focusing on the interplay between human and vector behaviours. To this end, we conducted a deep investigation of malaria vector biting rhythms over 16 h (from 16:00 to 08:00) and integrated the entomological data with key information about human sleeping patterns and net usage retrieved from a questionnaire submitted to village households.

## Methods

### Study area

The survey was carried out in Goden, a rural village (12°25ʹ N, 1° 21ʹ W) located in the central region of Burkina Faso in a Sudanese savannah area, 35 km east of the capital city of Ouagadougou. The region is characterized by holoendemic malaria mainly caused by *Plasmodium falciparum* (total malaria cases: 1,011,892; incidence: 354/1000 inhabitants [60, 61]) with a peak during the rainy season, which roughly starts in June and ends in October.

The village comprises around 145 compounds and is inhabited by approximately 900–1000 people (Bogodogo, Health District survey 2021, unpublished), mostly belonging to the Mossi ethnic group and devoted to agriculture and rearing of domestic animals (e.g. cows, pigs, dogs, goats and chickens) in their compounds. The village is also occasionally populated by settlements of Fulani, a historically nomadic ethnic group whose livelihood focuses on cattle rearing. As in the rest of the country, Goden has received a large and relatively stable supply of ITNs since 2010. Although specific data on the actual ITN coverage and usage in the study village are not available, the national survey *Enquête sur les indicateurs du paludisme au Burkina Faso* (2015 and 2018) [62, 63] reported that, in the Central region, about 55%, 86% and 79% of households received at least one ITN, respectively, in the first three distribution campaigns (2010, 2013 and 2016), with net usage between 55 and 60%. Roughly 3,800,000 ITNs were distributed during these campaigns, corresponding to about 96% household coverage (data unpublished, courtesy of Dr. Wamdaogo Moussa Guelbeogo).

### Entomological collections, specimen processing and molecular analysis

Host-seeking mosquitoes were collected by human landing catch (HLC) inside and outside three houses of three different compounds, from 16:00 to 08.00, for 8 nights from 9 to 25 September 2020. In each house, two volunteers rotated each hour between indoor and outdoor positions. Each couple of volunteers also rotated between compounds across the nights of sampling. During the sampling period, the volunteers performing the collections were the only human hosts present in the houses and no ITNs or any other form of vector control was used.

All collected mosquitoes were morphologically identified under stereomicroscope [64], separated by species and gender. A subsample of *Anopheles gambiae* s.l. females was selected to represent mosquito variability in each compound, house, date, position of samplings (indoors/outdoors) and collection time. The subsample was then processed as follows: heads-thoraces were separated from abdomens and individually stored in tubes containing desiccant until the DNA extraction by DNAZOL protocol (Molecular Research Centre, Cincinnati, OH, USA) [65]. Head-thorax extracted DNA was used as template for: (1) species identification by SINE-PCR protocol [66]; (2) real-time PCR genotyping of L1014F (kdr-w) mutation [67], the most common insecticide-resistance associated allele in the sodium-gated voltage-channel gene; (3) *Plasmodium* sporozoite DNA detection by real-time PCR [68]. To account for the potential contamination of head-thorax by an earlier infected blood meal, the abdomen of positive heads-thoraxes was also processed by PCR for *Plasmodium* DNA detection. Notably, it has been shown that molecularly detectable traces of *Plasmodium* can be still found in heads-thoraxes of 44% of experimentally infected females up to 6 days post-infection [69]. Therefore, a conservative calculation of sporozoite rate (SR) was applied under the assumption that the specimens analysed (all unfed) may have been infected in the last blood meal:

SR = HT + (0.56*HTAB)/*N* total specimens tested, where: HT is the number of *Plasmodium*-positive females in head-thorax only; HTAB is the number of *Plasmodium*-positive females in both head-thorax and abdomen; 0.56 is the corrective factor for *Plasmodium* positivity imputable to sporozoite stages in head and thorax. This factor is complementary to the 44% of specimens found infected in head/thorax by molecular traces of other life stages.

Finally, the entomological inoculation rate (EIR) of each species was calculated based on its abundance (GAM model described in “Statistical analyses”) and SR. The total EIR of malaria vectors was obtained as a sum of the EIR of each major species to account for their relative contribution to malaria transmission in the village. To estimate a more realistic EIR, considering actual human exposure to mosquito infective bites, we introduced a corrective factor calculated based on information on net usage and human habits retrieved from the questionnaires.

### Questionnaire

Representatives of 160 houses, organized in 80 compounds (two houses per compound), were anonymously interviewed by four technicians from the Centre National de Formation et Recherche sur le Paludisme (CNRFP) using a questionnaire designed to collect general information about bednet use and human habits. The interviews were carried out twice a month between 6:30 am and 9:30 a.m. from 5 October to 18 December 2020. Household representatives were interviewed once, apart from 16 who were mistakenly interviewed twice on different dates (5 October and 18 December).

The questionnaire comprised 24 questions about bednet presence, physical integrity and usage as well as human activities and number of inhabitants (Additional file 1: S1a); eight of these, used to fulfil the aim of the present work, were thematically split as follows:

Section A, questions about net availability and human sleeping habits.

- Q16. Do you have a bednet in the house?
- Q17. How many nets per person are present in the house?
- Q22. At what time do you go to sleep?
- Q23. At what time do you usually wake up?

Section B, questions about the number of inhabitants sleeping in the house and net usage the night before the interview.

- Q3. How many people slept in the house last night?
- Q2. How many children?
- Q6. How many people slept under a bednet? (i.e. number of protected people)
- Q7. How many people used the same bednet?

The questionnaire was translated from English to French, one of the official languages of Burkina Faso, to facilitate the investigators’ understanding of the text. For the houses interviewed twice, a mean value was considered for the answers given in Section B, as the number of people sleeping in a house can vary among different nights [70]. The answers to Q22 and Q23 were instead considered independently, since slightly different bedtimes and wake-up times were given between different dates of survey.

The number of unprotected people in each household was estimated by subtracting the “number of people that have slept under a net” (Q6) from the “number of people that have slept in the house” (Q3). In this process, the answers given to Q17, “number of available nets per inhabitants”, and to Q7, “number of people sleeping under the same net”, were considered as control questions. For some questionnaires (32 out of 176), the estimated number of unprotected people was considered unreliable, because the number of available nets was too low with respect to the number of people sleeping in the house. For these houses, the number of unprotected people was estimated assigning a range of minimum and maximum values. The percentage of unprotected people was then proportionally added to the awake households in each hour to estimate, respectively, the minimum and maximum “corrective factor” by which to adjust the EIR. This was done to estimate the actual EIR on the base of net usage and human sleeping patterns in the village.

The number of nets present in each house was extrapolated from question Q17, also considering the total of people sleeping in the house (Q3), the number of people that have slept under a net (Q6) and the number of unprotected people (estimated as previously described).

### Statistical analyses

A negative binomial generalised linear model (GLM) was fitted to the total number of *A. gambiae* s.l. females collected during the sampling period to estimate the average number of mosquitoes that were present in the 16-h sampling, position (IN/ OUT), house (1–3) and date of collection (see Additional file 2).

To investigate biting rhythms at species level, a generalized additive model (GAM) was fitted to the number of females for each species collected hourly by HLC indoors (IN) and outdoors (OUT) in each time frame (see Additional file 3). Since only a subsample of specimens was identified at the species level, the proportion for each species in the analysed subsample was used to estimate the overall number of females in each time frame.

Given the discrete nature of the response variable, in the case of over-dispersed data, a negative binomial was chosen as reference distribution in both GLM and GAM models.

GLM structure was:

$${Y}_{i}=\,{\beta }_{0 }+{\beta }_{1k }{[\mathrm{house}]}_{ki}+{\beta }_{2j} {[\mathrm{position}]}_{ji }+{\beta }_{3h} {[\mathrm{date}]}_{hi }+{\beta }_{4z} {[\mathrm{hour}]}_{zi }+{\varepsilon }_{i}$$

where ${Y}_{i}$ is the total number of collected mosquitoes, ${\beta }_{0}$ is the model intercept, ${\beta }_{1-4}$ represents the parametric coefficient for the linear effect, ${\left[\mathrm{house}\right]}_{k}$ represents a factor variable with three levels (the three sampled houses, *k* = 1,2,3), ${[\mathrm{position}]}_{j}$ is a dummy variable with two levels (samples collected inside or outside the houses, *j* = 1,2), ${[\mathrm{date}]}_{h}$ represents the date of the sampling (factor with 8 levels, *h* = 1,…,8), and ${[\mathrm{hour}]}_{z}$ represents the time interval of the collection (*z* = 1,…,16), and $\varepsilon$ is the error term for the *i*-th observation.

GAM model structure was:

$${Y}_{i}=\,{f}_{ihj}\left({[\mathrm{hour}]}_{i}* {\left[\mathrm{species}\right]}_{h}*{[\mathrm{position}]}_{j}\right)+{{\beta }_{hj}[\mathrm{species}]}_{h}*{[\mathrm{position}]}_{j}+ {\varepsilon }_{i}$$

where *Y*_*i*_ is the rounded proportion of mosquitoes collected, $f$ is a smooth function estimated using penalized likelihood maximization (with a smooth parameter estimated by restricted maximum likelihood) [71] for the *h*-th species in the *i*-th position; ${\left[\mathrm{position}\right]}_{j}$ represents a dummy variable with two levels (samples collected inside or outside the houses, *j* = 1,2) and ${\left[\mathrm{species}\right]}_{h}$ is also a factor with two levels (*h* = 1,2). Finally, ${\beta }_{hj}$ is the model’s parametric coefficient for the combined linear effect of the *j*-th position and the *h*-th species, while $\varepsilon$ is the error term for the *i*-th observation.

Chi-square test was employed to investigate differences in the sporozoite rate and L1014F mutant allele frequency between the species of the complex.

Finally, descriptive statistics were employed to extrapolate average values for each question in the questionnaire, obtaining some general information about net usage and human habits in the village.

## Results

### Species identification, infectivity rate and L1014F mutation genotyping

During the 8 nights of sampling, 15,201 *A. gambiae* s.l. females were collected landing on human volunteers. In the subsample of 1070 molecularly identified specimens, 68.7% were *Anopheles coluzzii* (*A.co*; *N* = 735); 26.2% *A. arabiensis* (*A.ar*; *N* = 280); 3.7% *A. gambiae* s.s. (*A.ga*; *N* = 40); 0.3% and 0.2% *A.co/A.ar* (*N* = 3) and *A.co/A.ga* (*N* = 2) hybrids, respectively. Ten specimens (0.9%) were not successfully identified by PCR. Overall, the heads-thoraxes of 27 specimens were positive for *Plasmodium. falciparum*, 1 for a mixed infection (presence of both *P. falciparum* and *Plasmodium vivax/P. ovale/P. malariae*) and 1 for *P. vivax*, *P. ovale* or *P. malariae*. Of 27 specimens positive for *P. falciparum* in heads-thoraxes (*A. coluzzii:* 3.7%; *A. arabiensis:* 0.7%; *χ*^2^ = 5.38; *p* = 0.02), 17 were also positive in the abdomens. By applying the conservative approach described in Methods section, SRs of 2.7% and 0.5% were estimated for *A. coluzzii* and *A. arabiensis*, respectively (*χ*^2^ = 2.96; *p* = 0.09). No intra-specific differences were detected in corrected SRs indoors vs. outdoors (*A.co*: 3.0% indoors, 2.3% outdoors, *χ*^2^ = 0.11, *p* = 0.74; *A.ar*: 0% indoors, 0.9% outdoors, phi = 0.07, *p* = 0.51).

The frequency of L1014F mutation was 0.68 (*n* = 326) in the vector population, significantly higher in *A. arabiensis* (89%; *n* = 127) than in *A. coluzzii* (53%; *n* = 199) *(χ*^2^ = 88.06, *P* < 0.0001, Table 1).

**Table 1:** Target site pyrethroid resistance mutation in malaria vectors

	Total genotyped (*n*)	L1014F genotype			
		RR	RS	SS	freq. R
*Anopheles coluzzii*	199	61	90	48	0.53
*Anopheles arabiensis*	127	105	16	6	0.89

Genotype and allele frequencies of L1014F (point mutation in the voltage-gated sodium channel gene) calculated from *Anopheles coluzzii* and *A. arabiensis* females collected in Goden village. *RR* homozygous resistant, *RS* heterozygous, *SS*  sensitive (wild type), *freq. R* frequency of resistant allele

### Vector abundance, biting behaviour and entomological inoculation rate

Results highlight a significant effect of date, house and time of collection on *A. gambiae* s.l. abundance (GLM = 62% deviance explained; Additional file 2: S2a). However, the relative species proportion stayed consistent between sampling dates and houses (Additional file 2: S2b). No difference was detected between mosquito collections indoors and outdoors (Additional file 2: S2a).

**Fig. 1:**
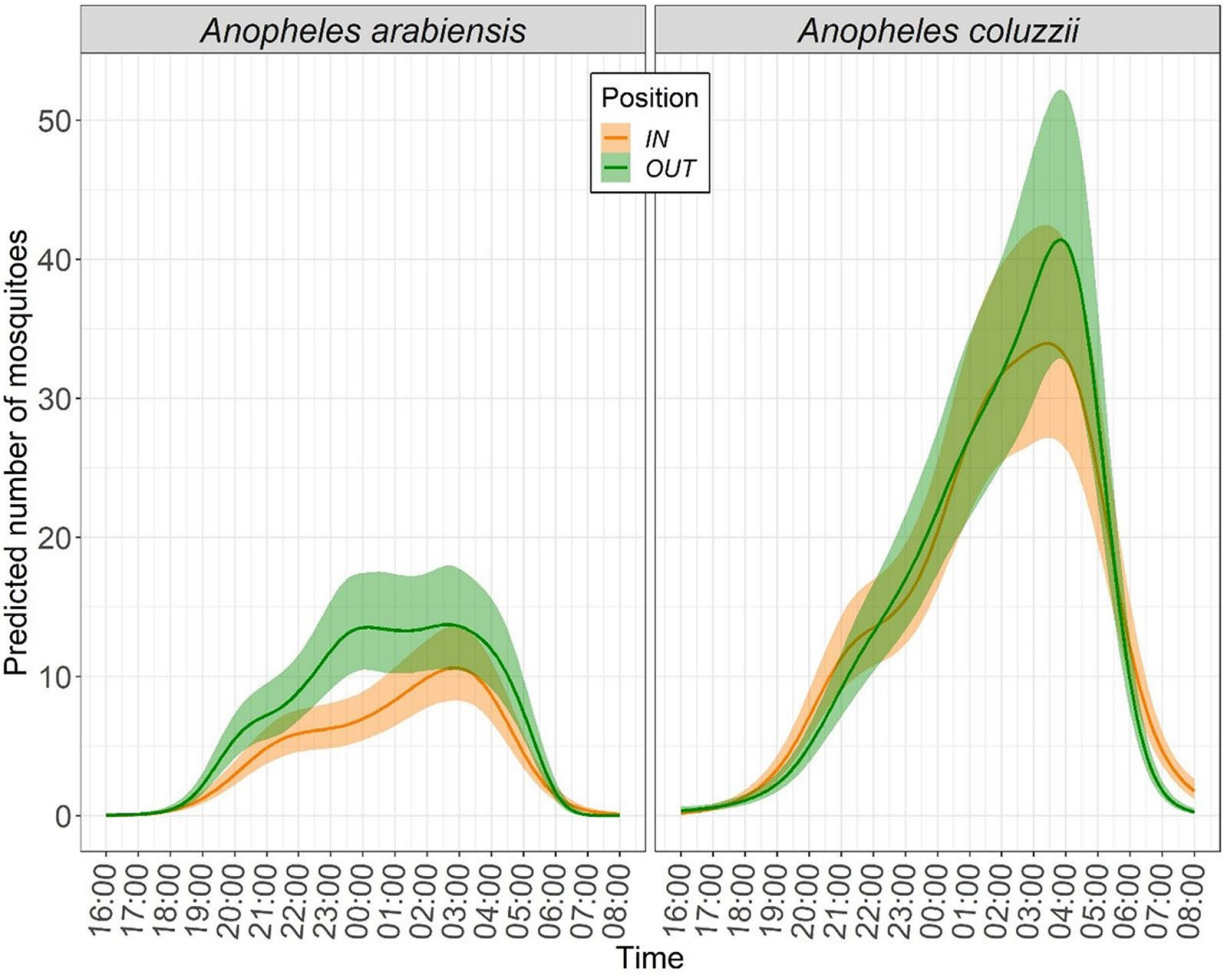
Hourly vector species biting activity. *Anopheles arabiensis* and *A. coluzzii* female temporal trends predicted by GAM throughout the 16 h of human landing catches carried out indoors (IN, red) and outdoors (OUT, green) in Goden village. The smooth graphs are derived from predicted coefficients of mosquito hourly abundances. The mean values and confidence intervals of the temporal trends are represented as continuous lines and dashed areas, respectively (see also Additional file 3: S3b)

A median of 14.4 and 5.4 mosquitoes/person/hour (m/*p*/h) is estimated during the 16-h sampling for *A. coluzzii* and *A. arabiensis*, respectively. Individual species analysis also highlights different temporal trends of biting activity for *A. coluzzii* and *A. arabiensis* (GAM 65.9% deviance explained; Table 2a, Fig. 1; see Additional file 3 for GAM output and 95% confidence intervals of hourly abundances). *Anopheles coluzzii* exerted an increasing biting pressure, with a peak between 00:00–04:00 and an equal activity between indoors and outdoors across most sampling hours, with the exception of the 19:00–21:00 and 06:00–08:00 periods, when the species is more abundant indoors (12.3 m/*p* indoors vs. 8.7 outdoors and 6.7 m/p indoors vs. 2.3 outdoors, respectively). Conversely, *A. arabiensis* shows a roughly homogeneous biting activity between 21:00 and 04:00, with a higher biting pressure exerted outdoors in most time points (i.e. 19:00–05:00: cumulatively 65.3 m/p indoors and 101.4 outdoors; 6:00–08.00: 0.5 m/p indoors and 0.08 outdoors).

**Table 2:** Hourly vector abundances and entomological inoculation rate based on human landing catches

(A)					(B)				
Hour	Mosquito female abundance (GAM estimated coefficients)				Entomological inoculation rate (EIR)				
	*Anopheles coluzzii* In	*Anopheles coluzzii* Out	*Anopheles arabiensis* In	Anopheles arabiensis out	*Anopheles coluzzii* EIR in	*Anopheles coluzzii* EIR out	*Anopheles arabiensis* EIR in	*Anopheles arabiensis* EIR out	Total hourly EIR (*A.co* + *A.ar*)
16–17 h	0.19	0.35	0.014	0.03	0.01	0.01	0.00	0.00	0.01
17–18 h	0.56	0.62	0.091	0.11	0.02	0.02	0.00	0.00	0.02
18–19 h	1.55	1.21	0.449	0.56	0.04	0.03	0.00	0.00	0.04
19–20 h	3.94	2.70	1.527	2.85	0.11	0.07	0.01	0.01	0.10
20–21 h	8.35	6.03	3.504	6.17	0.23	0.16	0.02	0.03	0.22
21–22 h	12.34	10.60	5.355	7.63	0.33	0.29	0.03	0.04	0.34
22–23 h	14.12	14.64	6.079	10.04	0.38	0.40	0.03	0.05	0.43
23–24 h	17.38	19.19	6.519	13.02	0.47	0.52	0.03	0.07	0.54
00–01 h	24.20	24.95	7.625	13.46	0.65	0.67	0.04	0.07	0.72
01–02 h	30.45	30.02	9.309	13.33	0.82	0.81	0.05	0.07	0.87
02–03 h	33.12	35.61	10.582	13.74	0.89	0.96	0.05	0.07	0.99
03–04 h	33.67	41.36	9.491	12.59	0.91	1.12	0.05	0.06	1.07
04–05 h	26.82	32.36	5.343	8.56	0.72	0.87	0.03	0.04	0.83
05–06 h	13.45	11.48	1.746	2.24	0.36	0.31	0.01	0.01	0.35
06–07 h	4.95	2.03	0.411	0.08	0.13	0.05	0.00	0.00	0.10
07–08 h	1.77	0.27	0.090	0.00	0.05	0.01	0.00	0.00	0.03
					Total *A.co* EIR (16 h)		Total *A.ar* EIR (16 h)		Total EIR (16 h)
					6.21		0.43		6.65

(A) GAM estimated coefficients of abundances for the main vectors species *Anopheles coluzzii* and *A. arabiensis* indoors (IN) and outdoors (OUT) throughout 16–h of sampling in Goden village. (B) *Anopheles coluzzii* and *A. arabiensis* hourly entomological inoculation rate (EIR) indoors and outdoors, calculated from species relative sporozoite rates (SR = 2.7% *A. coluzzii*; 0.5% *A. arabiensis*) and abundances. Total hourly EIR (A.co + A.ar) = estimated hourly cumulative EIR for the species *A. coluzzii* (A.co) and *A. arabiensis* (A.ar). Total EIR (16 h) = estimated EIR (indoors + outdoors) in the whole sampling period of HLC for *A. coluzzii* (A.co), *A. arabiensis* (A.ar) and cumulated for the two species

Considering the different abundance and infectivity of the two species, for the malaria vectors in the village an overall EIR of 6.7 infective bites/exposed person (mean of 0.4 infective bites/person/hour; Table 2b) throughout the 16 h-sampling period was estimated. This EIR corresponds to the maximum of malaria transmission risk for a human volunteer constantly exposed to mosquito bites throughout the biting period.

### Questionnaire and corrected EIR

Of the 176 questionnaires collected, 6 were excluded because of inconsistencies between the answers, probably because of a misunderstanding between the interviewer and the respondent (see Additional file 1: S1b for questionnaire limitations). The results here reported are retrieved from 170 questionnaires submitted to 157 house representatives (given that 16 of them were interviewed twice, see “Methods”) for an estimated population of 424 individuals. House representatives interviewed were 95% males and 5% females, all over the age of 22. Results of descriptive analyses reveal a median presence of two residents per house, with about 75% of houses composed of one to four inhabitants. Lack of nets is reported in only 3% of the houses, while in 97% at least one ITN is reported and 13% of houses had 2–3 nets. In 75% of houses, the same net was shared by up to two people, in line with the distribution policy [72]; in the rest of the houses, up to five people slept under the same net. Thus, based on the estimated number of inhabitants in the houses interviewed (*N* = 424), the percentage of unprotected people (i.e. people not using the net) ranged from 8.7 to 20.8%. Finally, 84% of respondents across the house (*N* = 170) mentioned going to sleep between 20:00 and 22:00 and 90% reported waking up between 5:00 and 7:00 (Table 3).

**Table 3:** Information on human habits used to estimate the hourly human exposure to vector bites

	Hour	*N* houses with people awake	% Houses with people awake	% Minimum human exposure (awake + minimum unprotected)	% Maximum human exposure (awake + maximum unprotected
Bed time	16–17 h	170	100%	100%	100%
	17–18 h	170	100%	100%	100%
	18–19 h	169	99%	99.1%	99.2%
	19–20 h	159	94%	94.5%	95.2%
	20–21 h	94	55%	58.9%	64.4%
	21–22 h	16	9%	16.9%	27.9%
	22–23 h	0	0%	8.7%	20.8%
	23–24 h	0	0%	8.7%	20.8%
	00–01 h	0	0%	8.7%	20.8%
	01–02 h	0	0%	8.7%	20.8%
	02–03 h	0	0%	8.7%	20.8%
	03–04 h	0	0%	8.7%	20.8%
Wake-up time	04–05 h	8	5%	13.3%	24.8%
	05–06 h	109	65%	68.0%	72.3%
	06–07 h	161	96%	96.3%	96.8%
	07–08 h	168	100%	100%	100%

Second column: number of houses in Goden village in which people declared being awake in each time frame; third column: percentage of houses with awake people in each time frame; fourth and fifth columns: percentage of human exposure in each time frame. These values have been estimated by proportionally adding, respectively, the minimum (8.7%) and maximum (20.8%) values of unprotected people to the percentage of houses with people awake

By combining the self-reported human sleeping patterns with the estimated percentages of unprotected people, we calculated minimum and maximum hourly human exposure rates (Table 3), which were then used as corrective factors of hourly EIR (Table 4).

**Table 4:** Corrected hourly entomological inoculation rates

Hour	Corrective factor (minimum)	Corrective factor (maximum)	Minimum cEIR	Maximum cEIR
16–17 h	1	1	0.01	0.01
17–18 h	1	1	0.02	0.02
18–19 h	0.991	0.992	0.04	0.04
19–20 h	0.945	0.952	0.09	0.10
20–21 h	0.589	0.644	0.13	0.14
21–22 h	0.169	0.279	0.06	0.09
22–23 h	0.087	0.208	0.04	0.09
23–24 h	0.087	0.208	0.05	0.11
00–01 h	0.087	0.208	0.06	0.15
01–02 h	0.087	0.208	0.08	0.18
02–03 h	0.087	0.208	0.09	0.21
03–04 h	0.087	0.208	0.09	0.22
04–05 h	0.133	0.248	0.11	0.21
05–06 h	0.680	0.723	0.24	0.25
06–07 h	0.963	0.968	0.10	0.10
07–08 h	1	1	0.03	0.03
			cEIR min (16 h)	cEIR max (16 h)
			1.23	1.95

Hourly minimum and maximum corrective factors and relative corrected entomological inoculation rates (cEIR). Last row reports the corrected EIRs estimated in 16 h of human landing catches conducted in Goden village

**Fig. 2:**
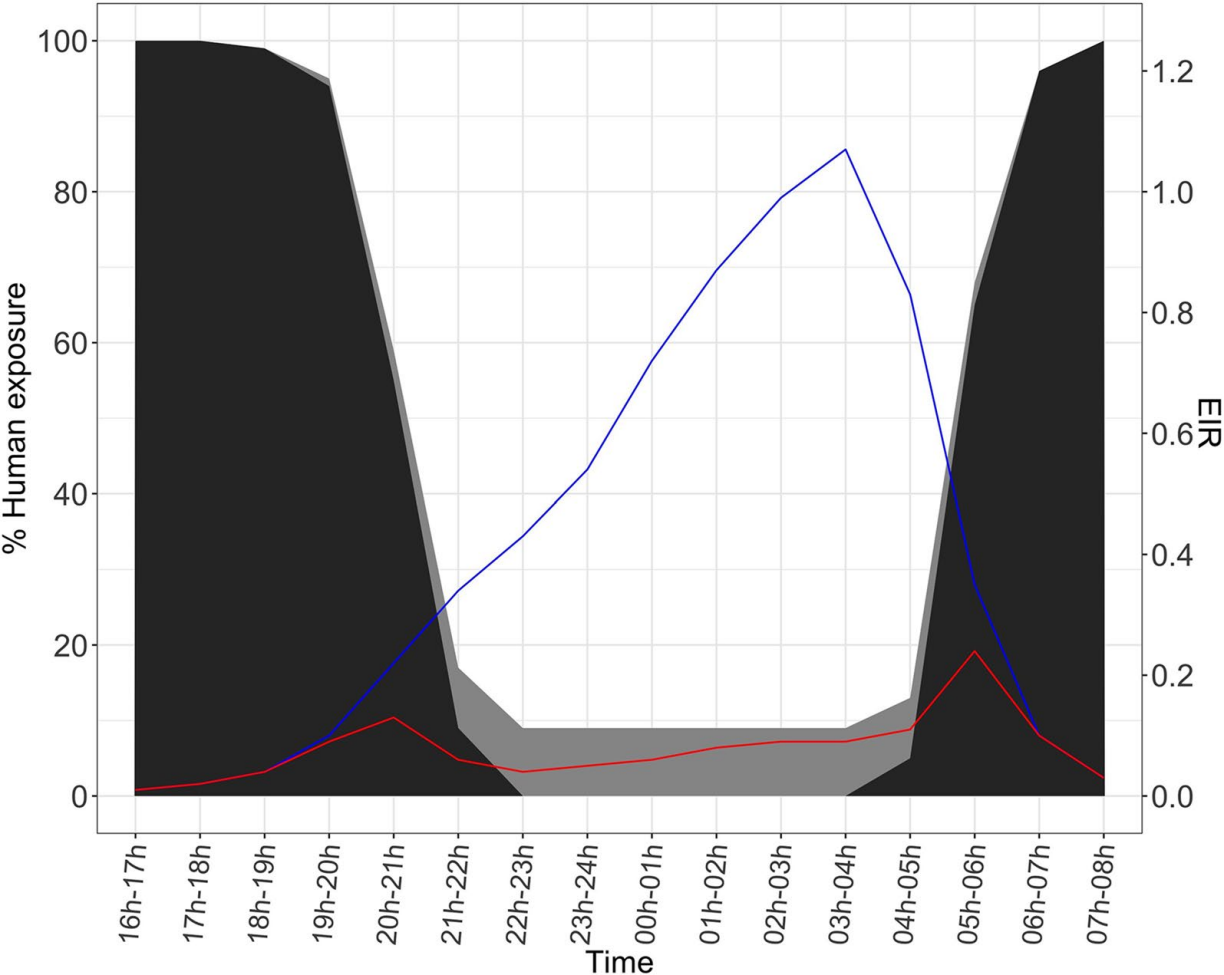
Hourly entomological inoculation rates and human exposure to bites. Entomological inoculation rates (EIR) and percentage of human exposure to malaria vector bites during the 16 h of human landing catches in Goden village. Shaded areas: proportion of subjects exposed to bites. This is calculated for each time frame by summing the proportion of houses with people awake (dark grey area) with the minimum proportion of subjects going to sleep without an ITN (i.e. unprotected; light grey area). Blue line: uncorrected EIR; red line: corrected EIR based on minimum human exposure level

The corrected estimated risk of malaria transmission during the 16-h sampling period ranges from 1.2 infective bites/person (when a minimum of 8.7% of people not using the net is considered, Fig. 2; Table 4) to 1.9 (when a maximum of 20.8% unprotected people is considered, Table 4). ITNs thus prevent at least 80% of malaria transmission risk, providing a fundamental contribution to individual protection since commonly used from 20:00 to 06:00 when biting pressure reaches an impressive high value of 30.3 mosquitoes/person/h (Table 2a). However, between 16:00–18:00 and 7:00–8:00 (namely when 100% of inhabitants are awake) mosquitoes have a cumulative diurnal biting activity of two bites/person (Table 2b), which accounts for a total of 0.06 infective bites/person (Fig. 2).

## Discussion

This study reports a persistent high malaria transmission risk in a village of Burkina Faso, despite over 10 years of ITN mass distribution. Compared to the previous HLC study conducted in the same village in 2015 (indoor/outdoor collections from 21:00 to 04:00) [58], a strong reduction in SR is observed in both *A. coluzzii* and *A. arabiensis*, likely due to an efficient individual protection exerted by ITNs (*A. coluzzii*: 2015 SR = 6%, 2020 = 2.7%; *A. arabiensis*: 2015 SR = 5.5%, 2020 SR = 0.5%). Nonetheless, the high mosquito biting rate observed in this study is indicative of a reduced killing effect of ITNs, which may compromise the community protection commonly expected when bednet coverage is > 50% [36, 73–76]. This threshold value is expected to be largely achieved, considering a median of two residents per house and that 95% of interviewed houses had at least one ITN.

Vector population in the village is dominated by *A. coluzzii*, which has the highest SR, consistent with its intense biting pressure during the entire sampling period (from 16:00 to 8:00) and its well-known anthropophilic preferences [10, 11, 35, 39, 77]. As opposed to *A. coluzzii*, *A. arabiensis* shows a lower SR and biting density, indicating its role as secondary malaria vector in the village. This is congruent with the infectivity levels already detected for *A. arabiensis* in the study area in previous reports [39, 58] as well as with literature evidence describing the generalist host choice behaviour of this species [11, 36, 39, 78–82]. Nevertheless, *A. arabiensis* should not be considered a vector of negligible importance, since its plastic biting behaviour makes it a resilient species to vector control tools, as shown by its widely recognised role in maintaining residual transmission in many East sub-Saharan countries, where other major anthropophilic vectors (i.e. *A. gambiae* and *A. funestus*) are strongly affected by indoor vector control interventions [38, 45, 55, 74, 83–91].

The results from this study do not conclusively indicate a marked alteration of biting rhythms—e.g. diversion toward early/late peaks—in response to net presence for *A. coluzzii* or *A. arabiensis*. In fact, *A. coluzzii* shows an increasing biting pressure up to 04:00 (although not significantly between 00:00 and 04:00, see Additional file 3: S3b), which was not observed in 2015 in the same village (when homogeneous biting activity was observed from 21:00 to 04:00, [58]) or in two other studies conducted after ITN implementation in Burkina Faso and Bioko Island [49, 50]. Conversely, *A. arabiensis* does not show a biting peak during central hours of the night (consistently with observations in 2015) [58], but this cannot be clearly associated to bednet presence, given the heterogeneous biting patterns reported for this species from other settings before and after ITN introduction [7, 41–43, 45, 92–95]. A slight preference for outdoor biting is observed only for *A. arabiensis*, while *A. coluzzii* does not exhibit any strong choice for biting location, as also observed in 2015.

Finally, from the epidemiological perspective, due to the dominance of *A. coluzzii* over *A. arabiensis* during the sampling period (end of rainy season), the dynamics of malaria transmission risk in the village is manly driven by *A. coluzzii* infectivity rates and biting patterns. In absence of protection by bednets, this implies that the probability for an exposed human host to receive a bite from an infective mosquito is higher during the central part of the night and does not vary indoors versus outdoors throughout the 16-h sampling period.

The association of entomological data and questionnaires on self-reported human behaviour throughout the mosquito biting period enables an integrated interpretation of malaria transmission dynamics in the context of ITN coverage. This approach indicated that the highest vector biting pressure is still exerted in the hours when humans are sleeping indoors (20:00–06:00), as observed in other regions of Burkina Faso [96] and other sub-Saharan countries [12, 57, 97, 98]. However, some studies showed marked diversion in biting time toward dusk or dawn after ITN/indoor residual spraying intervention indicating a variability in vector response according to geographical context [33, 41, 43, 46–48, 52, 56, 99–104]. The very high density of biting mosquitoes (around 20 females/person/hour) and the lack of a marked evasive behaviour in response to the net presence (early/late biting peak and mostly outdoor biting) are probably due to physiological resistance mechanisms to pyrethroid insecticides existing in the two species. The presence of phenotypic pyrethroid resistance in the overall vector population, although not directly recorded in this study, is evidenced by a high frequency of L1014F kdr resistance allele, which is the most widely screened target site resistance mutation. However, the level of L1014F allele frequency observed in this study is higher in *A. arabiensis* than in *A. coluzzii*. A longitudinal analysis of this mutation in Goden (from year 2011 to 2020; Perugini et al., unpublished) reveals opposite trends of frequencies in the two species, decreasing in *A. coluzzii* (from 0.72 to 0.56; *χ*^2^ = 38.74; *p* < 0.0001) and increasing in *A. arabiensis* (from 0.39 to 0.89; *χ*^2^ = 187.54, *p* < 0.0001). This suggests that L1014F frequency reduction in *A. coluzzii* can be compensated by the introduction of other target-site mutations [105] and/or that other different physiological resistance mechanisms can occur independently between the two species. Moreover, some forms of behavioural resistance/plasticity can contribute to further reduce bednet insecticidal pressure in both *A. coluzzii* and *A. arabiensis* [21, 33, 56], as in the case of the flexibility in host choice previously observed in Goden [39]. Collectively, this also results in a non-negligible human biting pressure (2 b/p) in the hours when people are awake (i.e. 16:00–18:00 and 7:00–8:00) and thus not protected by bednets, according with questionnaire results.

Results also highlight a dramatic gap in human protection that can be hard to minimise, even considering a scenario of full net coverage and accounting only for the human sleeping patterns. In fact, during the 16-h sampling, the exposure of awake inhabitants led to a risk of 0.69 i/b/p (i.e. 20.7 infective bites/person/month). In total, 10.3% of malaria transmission cannot be prevented by full coverage with ITNs in this setting. This gap in protection provides a hard core of residual malaria transmission in Goden, probably concurring to sustain the high level of parasite circulation despite over 10 years of bednet usage. This non-preventable exposure to bites is certainly a crucial element contributing to reducing the chances to reach the threshold EIR value of one infective bite/person/year required to proceed toward a feasible elimination of the disease [38, 106].

However, use of respondent-dependent methods, such as questionnaires, comes with embedded limitations. This is particularly true in our case, where the questionnaire was administered within an entomological study not tailored to describing human behaviour (extensively discussed in Additional file 1: S1b). In fact, individual data to quantify the temporal and spatial distribution of human exposure, as described by Monroe et al. [57], were not available. Our questionnaire relied on a single person being the respondent for all people of the household; in addition, no information was collected about the time spent indoors by inhabitants outside the ITN. Moreover, the proportion of unprotected people was obtained triangulating answers about net usage, number of inhabitants and number of available nets in each house. This approach did not allow the capture of micro-level behaviours (such as getting in and out of bed, removing an ITN or moving outdoors during the night) which combine to create additional gaps in protection potentially increasing the exposure to infective bites. Therefore, our measure of human exposure to mosquito bites is likely to be an underestimation. Nevertheless, this questionnaire offered the opportunity to extrapolate some reliable general information, e.g. range of unprotected people and human sleeping patterns, which are extremely useful in supporting interpretation of entomological data, and to assess an actual malaria transmission risk in the village. Finally, it is notable that the residual malaria transmission risk detected in the present study is likely to be affected by overlooked variables, from both anthropological and entomological perspectives. Indeed, in the study of Guglielmo and colleagues [107], the variables “gender”, “age” and “seasonality” had the greatest impact on the risk of human exposure to bites and it was shown that, even in the same week, this risk can vary up to 10 times depending on social events that lead people to be more active outdoors. Moreover, Sangbakembi-Ngounou and colleagues [108] have also described a non-negligible diurnal biting activity of malaria vectors (*A. gambiae*, *A. coluzzii*, *A. arabiensis* and *A. pharoensis*) indoors in an urban area of the Central African Republic. These studies highlight the difficulties in providing accurate estimations of residual malaria transmission risk and how, across different settings, the current vector control interventions are insufficient to eliminate transmission.

## Conclusions

In this study, a deep investigation across the mosquito biting window (16:00–08:00) has been paired with a survey on key information about human habits and net usage in a rural village of Burkina Faso. Results show that, although in the village the highest transmission risk is still occurring when most of the human population is protected under the nets, there is substantial residual malaria transmission—corresponding to 0.69 infective bites/person/day—which persists even considering full net coverage. This is paradigmatic of the problem of residual malaria transmission, which has been already extensively addressed by WHO [54] and tackled in some reviews and reports [55, 56, 109]. WHO emphasises the need to adopt integrated approaches to study malaria transmission dynamics and to generate additional evidence on the local residual malaria transmission. The magnitude of residual malaria transmission can vary greatly from one epidemiological context to another, according to both local entomological and anthropological factors [107, 108]; in some settings, the residual malaria transmission substantially limits the impact of current intervention strategies. Local tailored studies, as the current one in Goden, are essential to explore the heterogeneity of human exposure to infective bites and, consequently, to instruct the adoption of new vector control approaches [55, 110–112] by strengthening individual and community protection.

## Supplementary Information

**Additional file 1: S1.** a. Questionnaire, full version. b. Questionnaire limitations.

**Additional file 2: S2.** a. GLM output. b. Species abundances according to date and position of sampling.

**Additional file 3: S3.** a. GAM output. b. GAM predicted coefficients of *A. coluzzii* and *A. arabiensis* hourly abundances.

## Data Availability

Data supporting the conclusions of this article are included within the article and its additional files. The datasets used and analysed during the present study are available from the corresponding author upon reasonable request**.**

## Abbreviations

ITN: Insecticide-treated bednet
WHO: World Health Organisation
HLC: Human landing catch
SR: Sporozoite rate
HT: *Plasmodium*-positive females in head-thorax only
HTAB: *Plasmodium*-positive females in both head-thorax and abdomen
HBR: Human biting rate
EIR: Entomological inoculation rate
cEIR: Corrected entomological inoculation rate
CNRFP: Centre National de Recherche et de Formation sur le Paludisme
