# Supplementary material for: The interplay between malaria vectors and human activity accounts for high residual malaria transmission in a Burkina Faso village with universal ITN coverage
Source: Parasit Vectors. 2023 Mar 15;16:101. doi: 10.1186/s13071-023-05710-7 (PMC10015820; doi:10.1186/s13071-023-05710-7)
Supplement: Supplementary file 1 — Additional file 1: S1. a. Questionnaire, full version. b. Questionnaire limitations. [file 13071_2023_5710_MOESM1_ESM.docx]

**ADDITIONAL FILE 1**

**S1a: Questionnaire full version**

**QUESTIONNAIRE DE SUIVI DES CAPTURES AU PYRETHRE A GODEN**

Date Jour / mois / année I___I___I / I___I___I / I___I___I___I___I

Numéro de la concession I___I

1. **Questions relative à la nuit précédant la capture au pyrèthre**
2. Combien de personne habitent dans la maison ? I___I
3. Combien d’enfants habitent dans la maison ? I___I
4. Combien de personnes ont dormi la veille? I___I
5. A quelle heure les habitants reviennent à la maison ? I___I___I
6. Est-ce que tous habitants utilisent une moustiquaire imprégnée ?

Si I___I Oui, depuis quand ? année

Si I___I Non Pourquoi ?

1. Combien de personne utilisent une moustiquaire imprégnée ? I___I
2. Combien de personne dorment sous la même moustiquaire ? I___I
3. Les moustiquaires étaient-elles retirées ? Oui I___I ou Non I___I
4. Depuis combien de temps utilisent-ils les moustiquaires présentes dans la maison ?I___I
5. Qui leur a donner les moustiquaires ? I_________________________________________________________I

1. **Questions relative à l’utilisation des moustiquaires imprégnées**
2. Présence de trous sur la moustiquaire ?

Nombre I___I, Position : Coté 1à 4, et/ou toit : I___I ; Dimension : I___I (Pouce, Poinçon, tète).

1. Présence de moustiquaires nouvellement distribuées ? Marque I____I, Couleur I____I
2. Type de maison: Traditionnelle I___I, Moderne I___I
3. Type de matériaux de construction : Banco I___I, Ciment I___I, Bois I___I
4. Présence fenêtre ou ouvertures pour entrée des moustiques? Oui I___I ou Non I___I
5. **Questions générales**
6. Avez-vous une moustiquaire ? Oui I___I Non I___I
7. Nombre de moustiquaire par personne dans la maison : I___I/personne
8. A quelle fréquence utilisez-vous la moustiquaire pendant la saison pluvieuse ?

Tous les soir I___I, Souvent I___I, Rarement I___I, Jamais I___I

1. A quelle fréquence utilisez-vous la moustiquaire pendant la saison sèche ?

Tous les soir I___I, Souvent I___I, Rarement I___I, Jamais I___I

1. A quelle fréquence dormez-vous dehors ?

Tous les soir I___I, Souvent I___I, Rarement I___I, Jamais I___I

1. A quelle heure rentrez-vous habituellement à la maison ? I____I____I
2. A quelle heure allez-vous au lit ? I____I____I
3. A quelle heure réveillez-vous le matin ? I____I____I
4. Quand quittez-vous votre maison le matin ? I____I____I

Nom enqueteur__________________________________ Contact______________________

**S1b: Questionnaire limitations**

Limitations of the present survey both in questionnaire and question designs are here discussed.

The questions were not organized in a funnel system, from general to particular, and this might have increased the risk of inconsistences among the answers. In addition, while the questionnaire was written in French, the questions were formulated in Mòoré, one of the main languages of Burkina Faso and widely spoken in Goden village*.* Lack of a preliminary training to ensure a consistent and reliable translation from French to Mòoré meant that the language used by the interviewers might have altered the original meaning of the questions, reducing the pertinence of the answers and causing additional inconsistencies. The questionnaire design relied on one person being the respondent for each household. This means that factors such as recollection and social desirability bias (cf Choi & Pak Prev. Chronic. Dis.,2005) might have affected the answer: e.g., the respondent might not know the answer and yet be keen to provide one, providing the best possible answer from their perspective and not the most accurate from the perspective of the study. Specifically, this might be the case for answers about wake up and bed time, or about number of people sleeping under the same net.

During the data analysis, it became clear that some questions were leading for collector and/or respondent. This is the case of Q17 (number of nets available per inhabitants), which might have been unevenly translated and asked by the data collectors, partly due to the lack of pre-collection training mentioned above. Other questions offered the respondent a limited choice, not including the option “not known/not applicable” as a possible answer. This was the case of questions asking for a precise value, such as the “number of people sleeping under the same net” or “number of people who have slept in the house the night before the interview”.

Part of these limitations were minimized during the questionnaire analysis. For each questionnaire, answers were triangulated by using inner controls such as comparing responses given to similar or conceptually related questions, filtering out internally inconsistent questionnaires. For each house, answers were collected from only one individual, with the implicit understanding that their answers could be generalized. This understanding was optimistic, affecting data quality. Therefore, average values for all the respondents and households interviewed emerged as a better way of framing the responses received. At the end of the process, the questionnaire analysis had lost in detail but offered nonetheless the opportunity to extrapolate some reliable general information, such as range of unprotected people and timing of human activity, which are extremely useful factors in supporting entomological data interpretation.
