# Supplementary material for: The interplay between malaria vectors and human activity accounts for high residual malaria transmission in a Burkina Faso village with universal ITN coverage
Source: Parasit Vectors. 2023 Mar 15;16:101. doi: 10.1186/s13071-023-05710-7 (PMC10015820; doi:10.1186/s13071-023-05710-7)
Supplement: Supplementary file 2 — Additional file 2: S2. a. GLM output. b. Species abundances according to date and position of sampling. [file 13071_2023_5710_MOESM2_ESM.docx]

**ADDITIONAL FILE 2**

**S2a: GLM output**


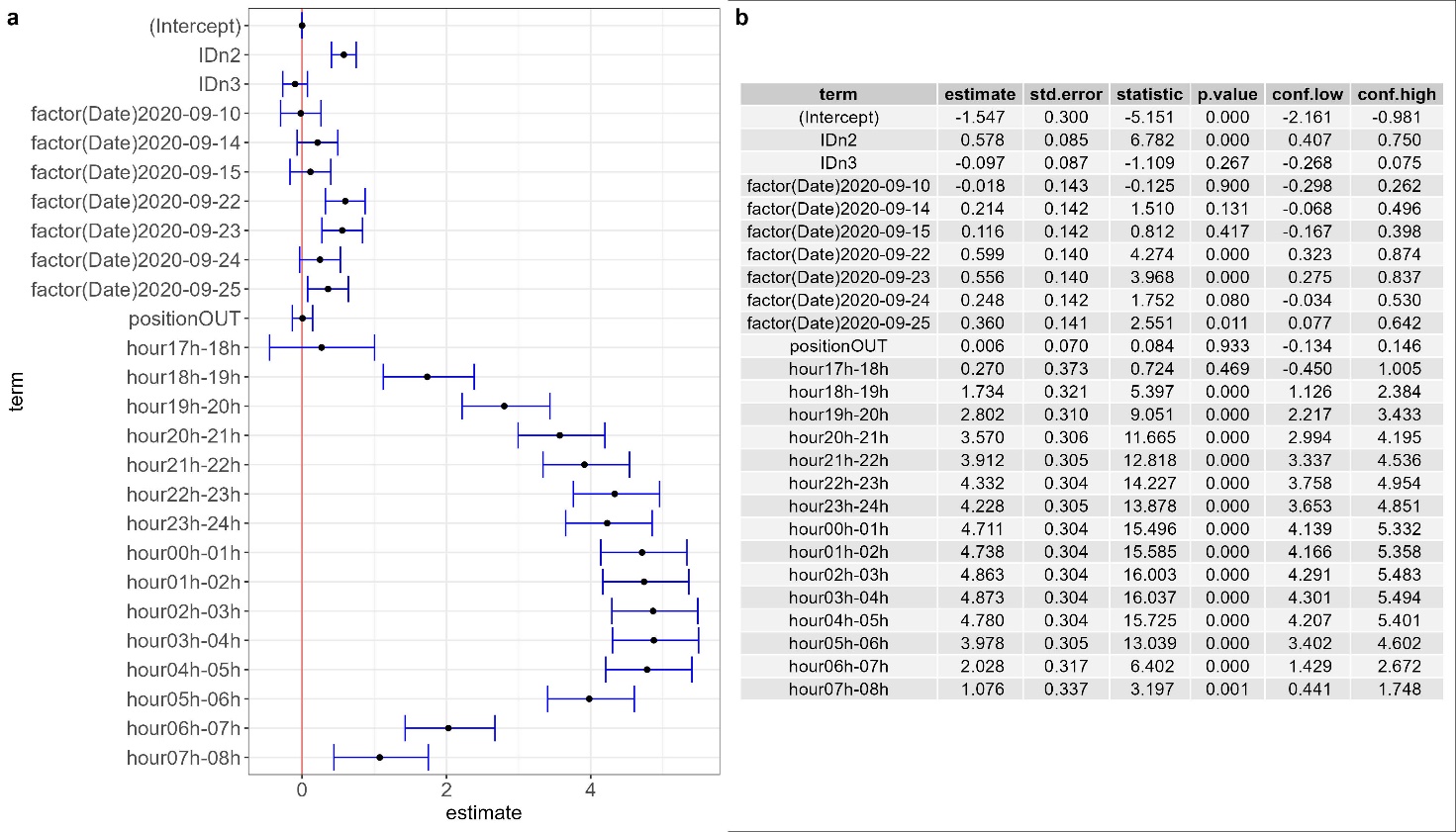
 GLM estimated coefficients. i) graphical representation of the coefficients: black dots represent the difference between the corner point (intercept) and the coefficients estimated by the model with respective confidence intervals (blue lines). The intercept was set to zero to make comparisons: if the confidence interval of the coefficient includes the zero value (red line), it means that there is no significant difference with the intercept. ii) table of the coefficients. Term: coefficient term; estimate: coefficient estimate for each term in the model, std.error: standard error; statistic: test statistic; conf.low: lower boundary of the confidence interval; conf. high: higher boundary of the confidence interval.

i)

ii)

**S2b: Species abundances according to date and position of sampling**

i)


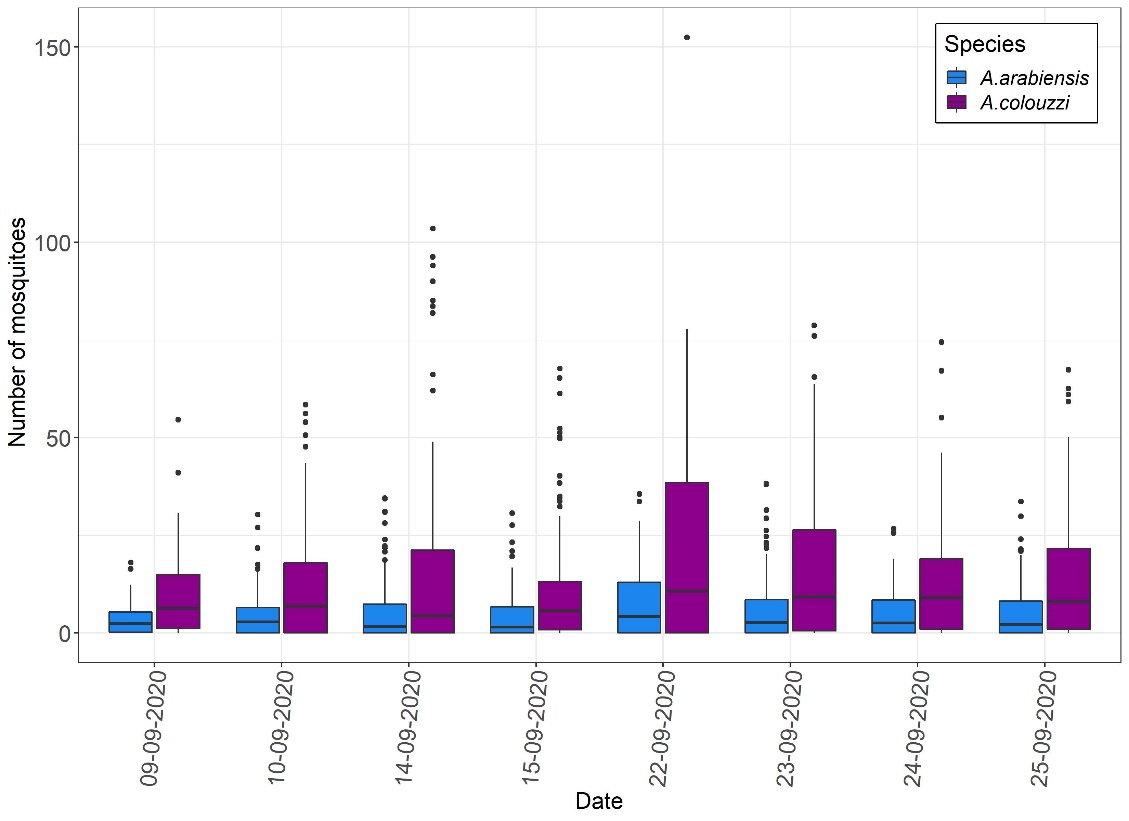


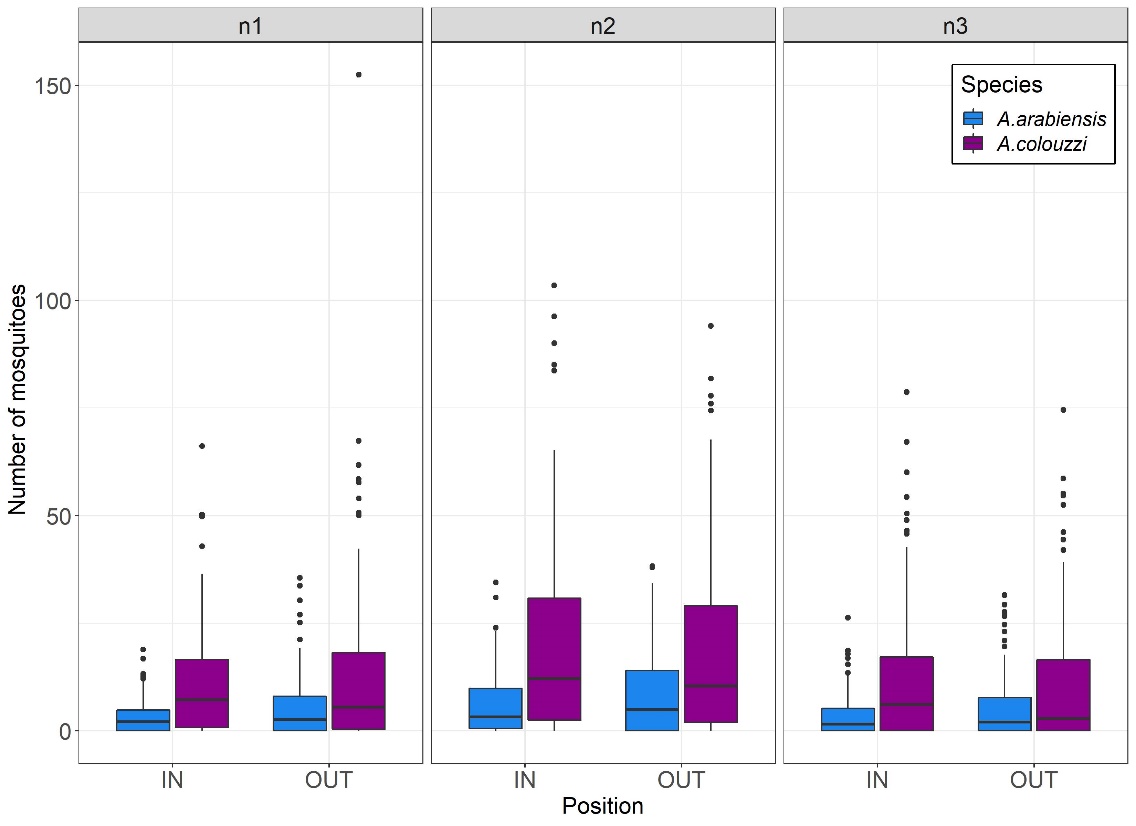


ii)

Numbers of A. coluzzii and A. arabiensis mosquitoes collected according to date (i) and position (ii). In the box plots, the boundary of the box closest to zero indicates the 25^th^ percentile, the black line within the box marks the median, and the boundary of the box farthest from zero indicates the 75^th^ percentile. The upper whisker extends from the hinge to the largest value no further than 1.5 x IQR from the hinge (IQR= inter-quartile range). The lower whisker extends from the hinge to the smallest value at most 1.5 x IQR of the hinge.
