# Supplementary material for: The interplay between malaria vectors and human activity accounts for high residual malaria transmission in a Burkina Faso village with universal ITN coverage
Source: Parasit Vectors. 2023 Mar 15;16:101. doi: 10.1186/s13071-023-05710-7 (PMC10015820; doi:10.1186/s13071-023-05710-7)
Supplement: Supplementary file 3 — Additional file 3: S3. a. GAM output. b. GAM predicted coefficients of A. coluzzii and A. arabiensis hourly abundances. [file 13071_2023_5710_MOESM3_ESM.docx]

**ADDITIONAL FILE 3**

**S3a: GAM output**


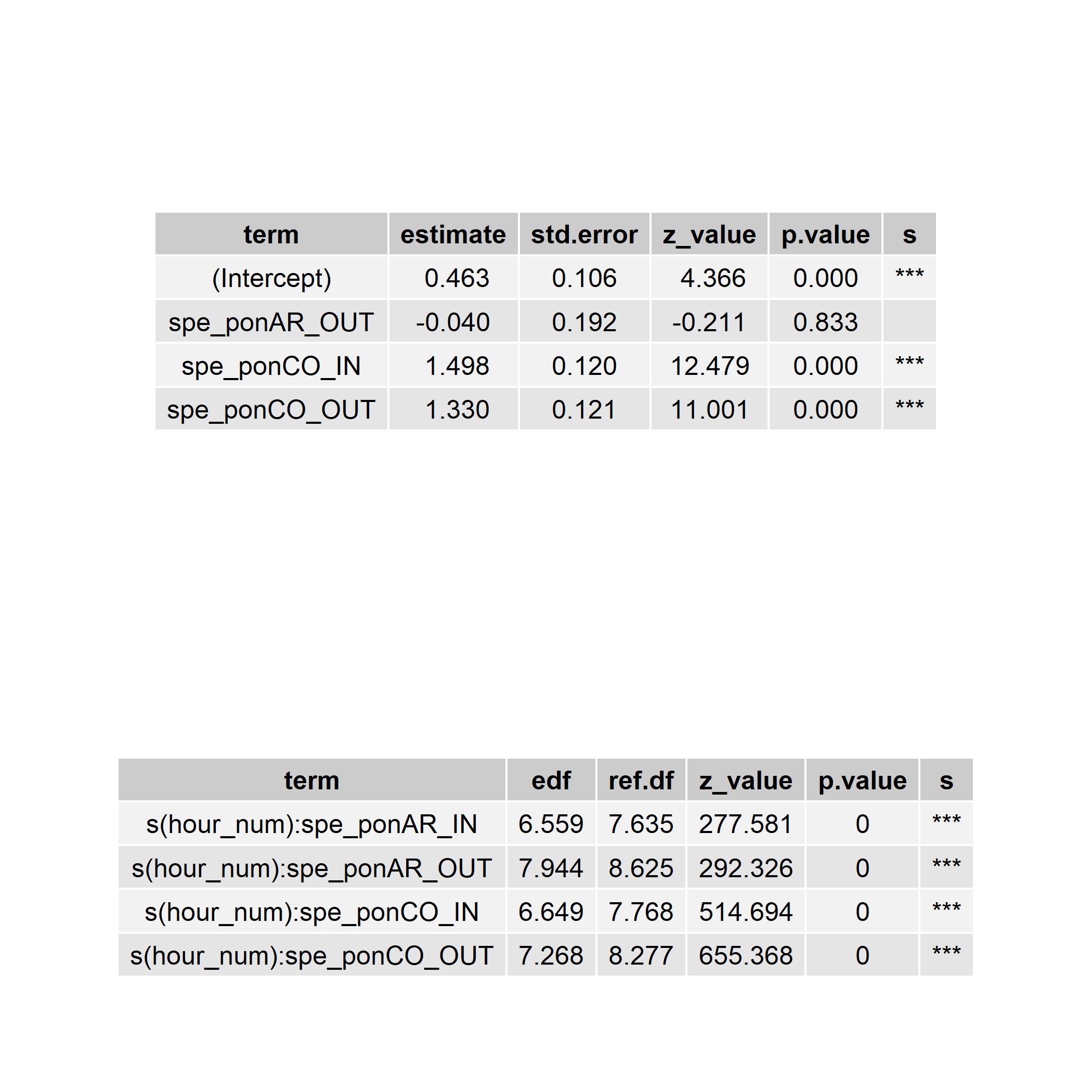


Table reporting the significance of the GAM smooth terms. Term: A. arabiensis (AR) and A. coluzzii (CO) species collected in relation to the position of sampling (IN and OUT); edf: effective degrees of freedom; ref.df: reference degrees of freedom; z-value: test statistic; s: significance.

| Hour | ***A.coluzzii* IN** | *A.coluzzii* IN  C.I. lower | *A.coluzzii* IN  C.I. upper | ***A.coluzzii* OUT** | *A.coluzzii* OUT  C.I. lower | *A.coluzzii* OUT  C.I. upper | ***A.arabiensis* IN** | *A.arabiensis*  IN  C.I. lower | *A.arabiensis*  IN  C.I. upper | ***A.arabiensis* OUT** | *A.arabiensis*  OUT  C.I. lower | *A.arabiensis*  OUT  C.I. upper |
| --- | --- | --- | --- | --- | --- | --- | --- | --- | --- | --- | --- | --- |
| **16h-17h** | **0.19** | 0.09 | 0.40 | **0.35** | 0.18 | 0.68 | **0.01** | 0.003 | 0.080 | **0.03** | 0.01 | 0.20 |
| **17h-18h** | **0.56** | 0.38 | 0.84 | **0.62** | 0.42 | 0.92 | **0.09** | 0.038 | 0.217 | **0.11** | 0.05 | 0.23 |
| **18h-19h** | **1.55** | 1.15 | 2.11 | **1.21** | 0.87 | 1.69 | **0.45** | 0.281 | 0.718 | **0.56** | 0.34 | 0.92 |
| **19h-20h** | **3.94** | 3.03 | 5.13 | **2.70** | 2.03 | 3.60 | **1.53** | 1.099 | 2.121 | **2.85** | 2.07 | 3.94 |
| **20h-21h** | **8.35** | 6.59 | 10.58 | **6.03** | 4.67 | 7.77 | **3.50** | 2.665 | 4.607 | **6.17** | 4.67 | 8.14 |
| **21h-22h** | **12.34** | 9.75 | 15.62 | **10.60** | 8.25 | 13.61 | **5.35** | 4.113 | 6.971 | **7.63** | 5.77 | 10.08 |
| **22h-23h** | **14.12** | 11.23 | 17.75 | **14.64** | 11.53 | 18.59 | **6.08** | 4.723 | 7.826 | **10.04** | 7.72 | 13.04 |
| **23h-24h** | **17.38** | 13.82 | 21.87 | **19.19** | 15.09 | 24.40 | **6.52** | 5.056 | 8.404 | **13.02** | 9.99 | 16.97 |
| **00h-01h** | **24.20** | 19.28 | 30.39 | **24.95** | 19.66 | 31.66 | **7.62** | 5.933 | 9.798 | **13.46** | 10.34 | 17.53 |
| **01h-02h** | **30.45** | 24.34 | 38.10 | **30.02** | 23.76 | 37.93 | **9.31** | 7.285 | 11.894 | **13.33** | 10.30 | 17.25 |
| **02h-03h** | **33.12** | 26.36 | 41.61 | **35.61** | 28.03 | 45.25 | **10.58** | 8.242 | 13.588 | **13.74** | 10.49 | 18.00 |
| **03h-04h** | **33.67** | 26.96 | 42.05 | **41.36** | 32.82 | 52.12 | **9.49** | 7.424 | 12.133 | **12.59** | 9.69 | 16.37 |
| **04h-05h** | **26.82** | 21.29 | 33.79 | **32.36** | 25.38 | 41.25 | **5.34** | 4.082 | 6.994 | **8.56** | 6.42 | 11.40 |
| **05h-06h** | **13.45** | 10.62 | 17.04 | **11.48** | 8.89 | 14.83 | **1.75** | 1.265 | 2.410 | **2.24** | 1.54 | 3.25 |
| **06h-07h** | **4.95** | 3.84 | 6.38 | **2.03** | 1.48 | 2.79 | **0.41** | 0.255 | 0.662 | **0.08** | 0.03 | 0.25 |
| **07h-08h** | **1.77** | 1.18 | 2.63 | **0.27** | 0.14 | 0.53 | **0.09** | 0.033 | 0.244 | **0.00** | 0.00 | 0.01 |

**S3b: GAM predicted coefficients of *A. coluzzii* and *A. arabiensis* hourly abundances**

GAM Estimated coefficients of A. coluzzii and A. arabiensis hourly abundances indoors (IN) and outdoors (OUT) and relative 95% Confidence Intervals (C.I.)
